# Structural basis for the tRNA-dependent activation of the terminal complex of selenocysteine synthesis in humans

**DOI:** 10.1093/nar/gkad182

**Published:** 2023-03-17

**Authors:** Anupama K Puppala, Jennifer Castillo Suchkou, Rachel L French, Kaitlyn A Kiernan, Miljan Simonović

**Affiliations:** Department of Biochemistry and Molecular Genetics, University of Illinois at Chicago, Chicago, IL60607, USA; Department of Biochemistry and Molecular Genetics, University of Illinois at Chicago, Chicago, IL60607, USA; Department of Biochemistry and Molecular Genetics, University of Illinois at Chicago, Chicago, IL60607, USA; Department of Biochemistry and Molecular Genetics, University of Illinois at Chicago, Chicago, IL60607, USA; Department of Biochemistry and Molecular Genetics, University of Illinois at Chicago, Chicago, IL60607, USA

## Abstract

*O*-Phosphoseryl-tRNA^Sec^ selenium transferase (SepSecS) catalyzes the terminal step of selenocysteine (Sec) synthesis in archaea and eukaryotes. How the Sec synthetic machinery recognizes and discriminates tRNA^Sec^ from the tRNA pool is essential to the integrity of the selenoproteome. Previously, we suggested that SepSecS adopts a competent conformation that is pre-ordered for catalysis. Herein, using high-resolution X-ray crystallography, we visualized tRNA-dependent conformational changes in human SepSecS that may be a prerequisite for achieving catalytic competency. We show that tRNA^Sec^ binding organizes the active sites of the catalytic protomer, while stabilizing the N- and C-termini of the non-catalytic protomer. Binding of large anions to the catalytic groove may further optimize the catalytic site for substrate binding and catalysis. Our biochemical and mutational analyses demonstrate that productive SepSecS•tRNA^Sec^ complex formation is enthalpically driven and primarily governed by electrostatic interactions between the acceptor-, TΨC-, and variable arms of tRNA^Sec^ and helices α1 and α14 of SepSecS. The detailed visualization of the tRNA-dependent activation of SepSecS provides a structural basis for a revised model of the terminal reaction of Sec formation in archaea and eukaryotes.

## INTRODUCTION

Synthesis and co-translational insertion of selenocysteine (Sec) is one of only two events in nature to expand the genetic code and incorporate a nonstandard amino acid into the proteome (1,2). Though resembling l-cysteine (Cys), Sec is distinct as it carries a selenol (SeH) moiety in place of a thiol. The comparatively lower pKa (5.2 versus 8.3) and redox potential (−488 mV versus −233 mV) of SeH render Sec fully ionized under physiological conditions (3,4), while its increased nucleophilicity causes Sec to be more reactive than Cys, thus arming selenoenzymes with both enhanced catalytic efficiencies (5,6) and resistance to oxidative inactivation (7). In higher organisms, selenoproteins and selenoenzymes play important biological roles and are pivotal for survival. Glutathione peroxidases and thioredoxin reductases remove reactive oxygen species and protect the cell membrane and DNA from oxidative damage (8–10), iodothyronine deiodinases maintain thyroid hormone homeostasis (11–13), and SelenoP regulates selenium (Se) levels (14–16). The systemic deletion of the cognate tRNA (tRNA^Sec^) is embryonically lethal in mice (17), and replacement of Sec with l-serine (Ser) or Cys compromises selenoenzyme activity and selenoprotein folding (18–20). Moreover, mutations and deficiency of selenoproteins cause disorders affecting various organ systems (21).

In contrast to the 20 canonical amino acids and pyrrolysine, there is no cellular pool of free Sec and the cognate SecRS never evolved (22–24). Instead, Sec synthesis occurs directly on tRNA^Sec^ in all organisms. The cycle commences with a misacylation event during which a promiscuous seryl-tRNA synthetase (SerRS) attaches Ser to tRNA^Sec^ (25), generating the first reaction intermediate, Ser-tRNA^Sec^ (26,27). In the subsequent steps, the bacterial and archaeal/eukaryotic Sec cycles diverge. Whereas the bacterial SelA directly converts Ser to Sec (28,29), archaea and eukaryotes employ l-seryl-tRNA^Sec^ kinase (PSTK) and *O*-phosphoseryl-tRNA^Sec^ selenium transferase (SepSecS) to improve the efficiency of SeH substitution (30). PSTK first activates the hydroxyl leaving group of Ser by ATP-dependent phosphorylation (31), and then SepSecS exchanges the phosphoryl group for SeH in a reaction dependent on mono-selenophosphate and a pyridoxal phosphate (PLP) co-factor (32,33). While many studies have helped elucidate these pathways, questions remain about how these enzymes distinguish tRNA^Sec^ and interact with one another to reliably generate Sec.

The evolution of both Sec pathways relied on specialized synthetic and translational machinery to form Sec on tRNA^Sec^ and recode an in-frame UGA stop codon (34). In all species, tRNA^Sec^ features structural elements distinct from canonical tRNAs that are central to the specificity, fidelity, and efficiency of the Sec synthetic enzymes. In contrast to the 7/5 acceptor-TΨC helix found in canonical tRNAs, tRNA^Sec^ adopts a longer 13-base pair (bp) acceptor-TΨC helix, resulting in an 8/5 fold in prokaryotes (35) and a 9/4 fold in archaea and eukaryotes (36). As the length of the acceptor arm impacts positioning of both the 5′-phosphate group and the CCA-end (37), its extension may influence productive interactions of tRNA^Sec^ with Sec-synthetic enzymes. Additionally, tRNA^Sec^ harbors enlarged D- and variable arms that could serve as auxiliary recognition determinants and/or anti-determinants. Moreover, the lack of otherwise conserved interactions between the 8th nucleotide of the acceptor arm and the D-arm may engender tRNA^Sec^ with some conformational malleability (38,39). This flexibility could allow productive interactions with SerRS while retaining specificity for SelA, PSTK, and SepSecS.

The divergence in the mechanisms of SeH substitution between prokaryotic and archaeal/eukaryotic systems is evident in the differences between SelA and SepSecS. Both enzymes are Fold Type I PLP-dependent enzymes with catalytic sites positioned at the dimer interfaces. Along with SepCysS, SelA and SepSecS are the only Type I PLP-dependent enzymes that act on a tRNA substrate, yet each of these enzymes occupy phylogenetically distinct branches (40). Whereas SelA is a functional homodecamer that binds up to 10 tRNA^Sec^ molecules (29), SepSecS is a tetramer (41). SelA primarily recognizes the extended D- and TΨC arms of tRNA^Sec^ (29), while SepSecS approaches tRNA^Sec^ from the opposite side where it establishes contacts with the variable arm and the minor groove of the acceptor arm (32). Early structural work revealed a cross-dimer substrate binding mode for complex formation wherein SepSecS is pre-ordered for binding and catalysis (42). Despite possessing four equivalent tRNA-binding and active sites, SepSecS only acts on up to two tRNA^Sec^ molecules at a time (43), leading to a half-sites occupancy. In this arrangement, one SepSecS dimer, designated the non-catalytic protomer, docks two tRNAs and situates the CCA-ends near the catalytic sites in the neighboring catalytic protomer. The other dimer is the catalytic protomer which establishes tRNA^Sec^ identity and provides sites for catalysis (32). Surprisingly, with the exception of minor side-chain rearrangements in the phosphate binding loop (P-loop), the catalytic and non-catalytic promoters largely resembled one another in crystal structures (32,43). Although previous studies indicated that SepSecS utilizes a tRNA-binding mechanism dissimilar to its closest orthologs (44), the structural elements in SepSecS and tRNA^Sec^ governing formation of the productive terminal complex remained poorly understood. Overall, the originally proposed model of SepSecS catalysis failed to explain the half-sites occupancy, the supposed pre-ordered conformation of the enzyme for catalysis, the absence of any substrate-induced conformational changes in SepSecS, and the mechanism whereby the enzyme senses leaving groups and reaction products.

To address these outstanding questions, we performed a thorough structural and biophysical analysis of the human holo SepSecS and SepSecS•tRNA^Sec^ binary complex. Our new high-resolution crystal structures reveal that tRNA binding induces a conformational change of the P-loop in the active sites of the catalytic protomer, while also stabilizing the extreme N- and C-termini of the non-catalytic protomer. The structural adjustment of the N-terminus allows the CCA-end of tRNA^Sec^ to access the active-site pocket, while the stabilization of the C-terminus may regulate the overall complex architecture. Furthermore, our data show that complex formation between SepSecS and tRNA^Sec^ is enthalpically driven and mediated by electrostatic interactions between helices α1 and α14 of the enzyme and the sugar-phosphate backbone of the acceptor-TΨC arms of tRNA^Sec^. Moreover, residues of α14 help establish the catalytically competent state of the binary complex. Altogether, this study clarifies how enzyme-substrate interactions mediate the specificity and formation of a catalytically competent complex, revising the paradigm for the terminal reaction of Sec synthesis in archaea and eukaryotes.

## MATERIALS AND METHODS

### Crystallization and data collection

Crystals were obtained by the vapor diffusion, sitting drop method in a 96-well plate format (Hampton Research). Prior to assembly, tRNA^Sec^ was heat denatured for 1 min at 90–95°C (20 mM Tris, pH 8.0, 150 mM NaCl) and allowed to cool to room temperature and renature on the bench. For crystallization, the holoenzyme and complexes were assembled in the assembly buffer: 20 mM Tris, pH 8.0, 200 mM NaCl, 5% (v/v) glycerol, 10 μM PLP, and either 0.5 mM TCEP (native SepSecS) or 5 mM TCEP (SeMet-SepSecS). Crystals of the holoenzyme were grown with 4 mg/ml human SepSecS mixed with 1 mg/ml unacylated tRNA^Sec^ in 0.36 M lithium citrate, 15% (w/v) PEG 3350, and 0.1 M sodium cacodylic acid titrated with 0.04 M HCl to pH 6.3. Crystals of the underivatized binary complex were grown using 2.5–7.5 mg/ml human SepSecS•tRNA^Sec^ (with a 2-fold molar excess of SepSecS) in 0.24 M lithium citrate, 9–10% (w/v) PEG 3350, and 0.1 M sodium cacodylic acid titrated with HCl to a pH of 6.2–6.4. Crystals of the SeMet-derivatized binary complex were produced with 5.0 mg/ml of human SeMet-SepSecS•tRNA^Sec^ (with a 2-fold molar excess of SepSecS) in 0.28–0.3 M ammonium acetate, 19.8% (v/v) MPD, and 0.1 M sodium citrate titrated with 0.057 M HCl to a pH of 5.5. For all setups, 1 μl of the protein or complex was mixed with 1 μl of reservoir buffer and crystals were grown at +12°C. After structure determination, we identified that the originally cloned SepSecS gene harbored a V491A mutation.

Crystals grown in the presence of PEG 3350 were cryoprotected with 20% (v/v) ethylene glycol prior to X-ray exposure, and those obtained with MPD were cryoprotected using 30% (v/v) MPD. The diffraction data were collected at cryogenic temperatures at the Life Sciences Collaborative Access Team (LS-CAT) beamline at APS-ANL. For the SeMet complex, a Se fluorescence spectrum scan in SepSecS-tRNA^Sec^ crystals indicated that wavelength of 0.979439 Å was optimal for anomalous diffraction data collection. The X-ray diffraction data were processed in HKL-2000 (45).

### Structure determination and refinement

The holo SepSecS structure was solved by molecular replacement using PDBID 3HL2 as a starting model and Phaser (46) within the Phenix software package (47). For the underivatized binary complex, the 3HL2 complex, in which tRNA^Sec^ is bound to the tetramer in two alternative conformations (32), was used as an initial model for refinement. The crystal structure of SeMet-SepSecS in complex with human tRNA^Sec^ was determined by single-wavelength anomalous diffraction (SAD) phasing based on SeMet. SHELX was used to determine the positions of 58 (including two alternate confirmations) out of a possible 68 Se atoms (48). To improve the phase estimate, several rounds of density modification in DM (49) were performed. Iterative model building and structure refinement were done in Coot (50) and Phenix (51), respectively. For the purposes of model building, the tRNA conformations were split into two separate molecules and then combined into one molecule for refinement. The occupancy for each conformation of tRNA^Sec^ during refinement was fixed at 0.5. All figures were made using PyMOL Molecular Graphics System, Version 2.4.2 Schrödinger, LLC.

### Structural analysis

To improve visualization of α16, feature-enhanced maps (FEM) that minimize noise and model bias were calculated using Phenix (52). The electrostatic potential surface for holo SepSecS and tRNA^Sec^ were calculated in PyMOL (version 2.4.2) with continuum electrostatic calculations using the Adaptive Poisson-Boltzmann Solver (APBS) software package plugin (53). Briefly, holo SepSecS was superimposed onto SepSecS complexed with tRNA^Sec^ in PyMOL. The holoenzyme and tRNA^Sec^ from the binary complex structure were converted to a PQR file using PDB2PQR. The PQR file was then analyzed by APBS using the default settings with a solvent probe radius of 1.4 Å, surface sphere density of 10 grid points/Å^2^. Temperature was set to 310 K, ionic strength to 0.15 M in monovalent salt, and the dielectric constants for solute (protein and ligands) and solvent to 2.0 and 78.00, respectively.

### Tycho unfolding profiles

Protein stability and integrity of the WT and SepSecS mutants were evaluated by comparing thermal unfolding profiles generated by a Tycho instrument (NanoTemper Technologies). For sample preparation, all proteins were diluted to 1 mg/ml (17.3 μM) in 20 mM HEPES, pH 8.0, 150 mM NaCl, 5% (v/v) glycerol, and 0.05% (v/v) Tween-20. The diluted protein samples were spun for 5 minutes at 12000 rpm to pellet and remove any aggregated protein. Finally, samples were loaded into Tycho capillaries (NanoTemper Technologies) and analyzed in duplicate.

### Micro-scale thermophoresis (MST) binding assay

To follow binding during MST, each SepSecS mutant was labeled using the Monolith Protein Labeling Kit RED-NHS 2nd Generation (NanoTemper Technologies). The labeling reaction was performed according to the manufacturer's protocol. Briefly, 20 μM of protein was mixed with the dye (in the supplied buffer), keeping a dye-to-protein molar ratio of 3:1 and incubated in the dark for 30 min. Unreacted dye was removed with the supplied, dye-removal column equilibrated with 20 mM HEPES, pH 8.0, 150 mM NaCl, 5% (v/v) glycerol, and 0.05% (v/v) Tween-20. The protein concentration and degree of labeling were determined using UV/VIS spectrophotometry at 650 and 280 nm. A degree of labeling of ∼0.8–1 was typically achieved. Subsequently, bovine serum albumin (BSA) was added to the labeled protein to a final concentration of 0.4 mg/ml.

For the MST experiment, the labeled SepSecS was adjusted to 10 nM with MST assay buffer (with 20 mM HEPES, pH 8.0, 150 mM NaCl, 5% (v/v) glycerol, 0.05% (v/v) Tween-20, and 0.4 mg/ml BSA). Prior to complex assembly, tRNA^Sec^ was heat denatured for 1 min at +90–95 °C (20 mM HEPES, pH 8.0, 150 mM NaCl) and allowed to cool to room temperature and renature on the bench. Dilution series were then prepared according to the MO.Control software-protocol (NanoTemper Technologies) generated from an estimated *K*_d_. A series of 2-fold dilutions of tRNA^Sec^ were prepared in 10 μl of MST assay buffer to yield a range of tRNA^Sec^ concentrations. For each measurement, 10 μl of each ligand dilution was mixed with 10 μl of labeled SepSecS, which led to a working SepSecS concentration of 5 nM. After 10 min, the samples were loaded into Monolith NT.115 Premium Capillaries (NanoTemper Technologies). MST for WT SepSecS was measured using the Monolith NT.Automated (NanoTemper Technologies) using 15% LED power and medium MST power. All other measurements were performed on a Monolith NT.115Pico instrument (NanoTemper Technologies) at room temperature using 5% LED power and medium MST power.

For the R398A and R398E mutants of SepSecS and Mut5 of tRNA^Sec^, the setup was adjusted to maximize the ligand concentration. A series of 2-fold dilutions of tRNA^Sec^ were prepared in 20 μl, and then 18 μl of each ligand dilution was mixed with 2 μl of 50 nM labeled SepSecS. The tRNA^Sec^ concentrations were input into the MO.Control software and run in Expert Mode, using 5% LED, medium MST power, and an MST on-time of 20 s. For all studied interactions, replicates (*n = 3–6*) from independently pipetted measurements were analyzed (MO.Affinity Analysis software version 2.3) using the signal from a 5 s MST on-time.

### van’t Hoff calculations

To determine the enthalpy and entropy of binding between SepSecS and tRNA^Sec^, MST for a single set of capillaries was run at +24, +26, +28, +30, +32 and +34°C to determine the *K*_d_ for the same sample at each temperature. Replicates of five or six per species were run using 5% LED power and medium MST power and analyzed using an MST on-time of 5 s. From the temperature and the associated *K*_d_ value, we generated the corresponding van’t Hoff plot by plotting ln(*K*_a_) versus 1/*T* (54). A linear regression of the data (Equation 1) determined the slope and *y*-intercept, allowing calculation of the enthalpy (Δ*H*°) and entropy (Δ*S*°) of binding according to Equation (1):


(1) $$\begin{equation*}{\rm ln}\left( {{K}_a} \right) = \ - \frac{{\Delta H^\circ }}{{RT}}\ + \ \frac{{\Delta S^\circ }}{R}\end{equation*}$$

### *E. coli* SepSecS complementation assay

The activity of WT and the human SepSecS variants was assessed by evaluating their ability to rescue the loss of SelA in Δ*selA* JS2(DE3) cells via the activity of the selenoenzyme, formate dehydrogenase (FDH) (55). The day prior to the assay, we inoculated LB broth supplemented with 1% (w/v) glucose, carbenicillin (100 μg/ml), and chloramphenicol (34 μg/ml) and grew each strain aerobically for 16 h at +37°C. Cells were centrifuged and resuspended in sterile PBS to a cell density of 4 × 10^9^ cells/ml. Each strain was then serially diluted in PBS to a cell density of 4 × 10^5^ cells/ml. Subsequently, 10 μl of each dilution series was plated onto a row of square LB agar plates containing carbenicillin (100 μg/ml), chloramphenicol (34 μg/ml), 10 μM IPTG, 1 μM Na_2_MoO_4_, 1 μM Na_2_SeO_3_, 50 mM HCOONa and 0.5% (w/v) glucose. On a separate LB plate for downstream validation experiments, 250 μl of each undiluted culture was plated. Plates were incubated in an anaerobic chamber (Type A vinyl 110V, Coy Lab Products) with a gas mix of 90% N_2_, 5% H_2_, 5% CO_2_ for 24 h at +25°C. The next day, the LB top agar (0.75% (w/v) agar) was prepared and supplemented with 1 mg/ml benzyl viologen (BV), 250 mM HCOONa and 25 mM KH_2_PO_4_ (pH 7.0). For each assay plate, 10 ml of the supplemented top agar was poured on and gently distributed to cover the plate. To visualize the BV reduction, plates were imaged 30 min after the overlay with the top agar.

## RESULTS

### High resolution structures of holo SepSecS and SepSecS in complex with unacylated human tRNA^Sec^

Optimization of crystallization conditions and purification protocols (32,42) improved the diffraction quality of crystals containing either holo SepSecS, native SepSecS•tRNA^Sec^, or SeMet-derivatized SepSecS•tRNA^Sec^. Crystals of holo SepSecS diffracted to 2.25 Å, whereas the native and SeMet binary complex crystals diffracted to 2.32 and 2.07 Å resolution, respectively (Supplementary Table S1). Diffraction power at higher angles of binary complex crystals grown in the presence of PEG 3350 was of limited quality. In contrast, the complex crystals obtained from MPD-containing buffers consistently yielded well-defined reflections in higher resolution shells, thus permitting SAD phasing experiments. The final maps were of outstanding quality, allowing construction of the most comprehensive models of human SepSecS to date. Experimental SAD phases showed strong peaks in the anomalous difference maps (Supplementary Figures S1A, B), which allowed positioning of Se atoms in SepSecS and provided an additional layer of confidence for structural analysis (Supplementary Figures S1C-E). It is prudent to mention we later discovered that the SepSecS used for crystallization harbored an inadvertent Val491Ala mutation. The mutation was corrected for all downstream experiments, and given the similarity between Ala and Val, this mutation is unlikely to have any effect on the structural results and interpretations.

**Figure 1.**
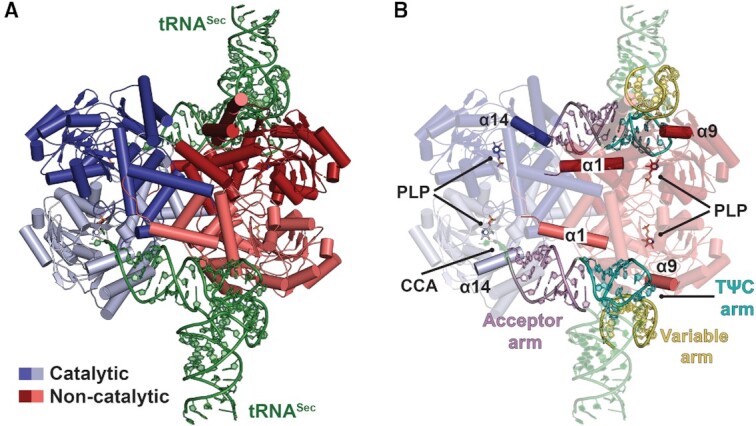
Architecture of the terminal complex of Sec synthesis in humans. (**A**) Cartoon representation of the binary SepSecS•tRNA^Sec^ complex. The SepSecS catalytic protomer is in shades of blue, the non-catalytic protomer in shades of red, and tRNA^Sec^ is green. (**B**) SepSecS employs helices α1 and α14 to engage the acceptor and TΨC arms (pink and cyan cartoon, respectively) and helix α9 to contact the variable arm (yellow cartoon) of tRNA^Sec^.

Structural superimposition of SepSecS tetramers derived from our structures yielded RMSD values within ∼0.4 Å (Supplementary Figure S2), establishing that human SepSecS adopted the same quaternary structure in all crystal forms. Both binary complexes exhibited a common tetrameric architecture with the enzyme binding tRNA^Sec^ in a cross-dimer fashion (Figure 1A). The primary binding elements mediating complex formation are helices α1, α9, and α14 of SepSecS and the acceptor-TΨC and variable arms of tRNA^Sec^ (Figure 1B). The non-catalytic protomer employs helices α1 and α9 to dock the acceptor-TΨC and variable arms of tRNA^Sec^, whereas conserved regions of α14 in the catalytic protomer position the 3′-end of the acceptor arm of tRNA^Sec^ near the catalytic groove.

### tRNA^Sec^ binding induces conformational changes in the non-catalytic SepSecS protomer

Previous structures suggested that SepSecS adopted a fold pre-ordered for tRNA^Sec^ binding (32), with binding-induced conformational changes occurring only in the tRNA substrate. Yet, such a model could not explain how the enzyme recognizes substrate binding to initiate catalysis nor perceives product formation for release after catalysis. New high-resolution crystal structures allowed us to further probe these questions.

**Figure 2.**
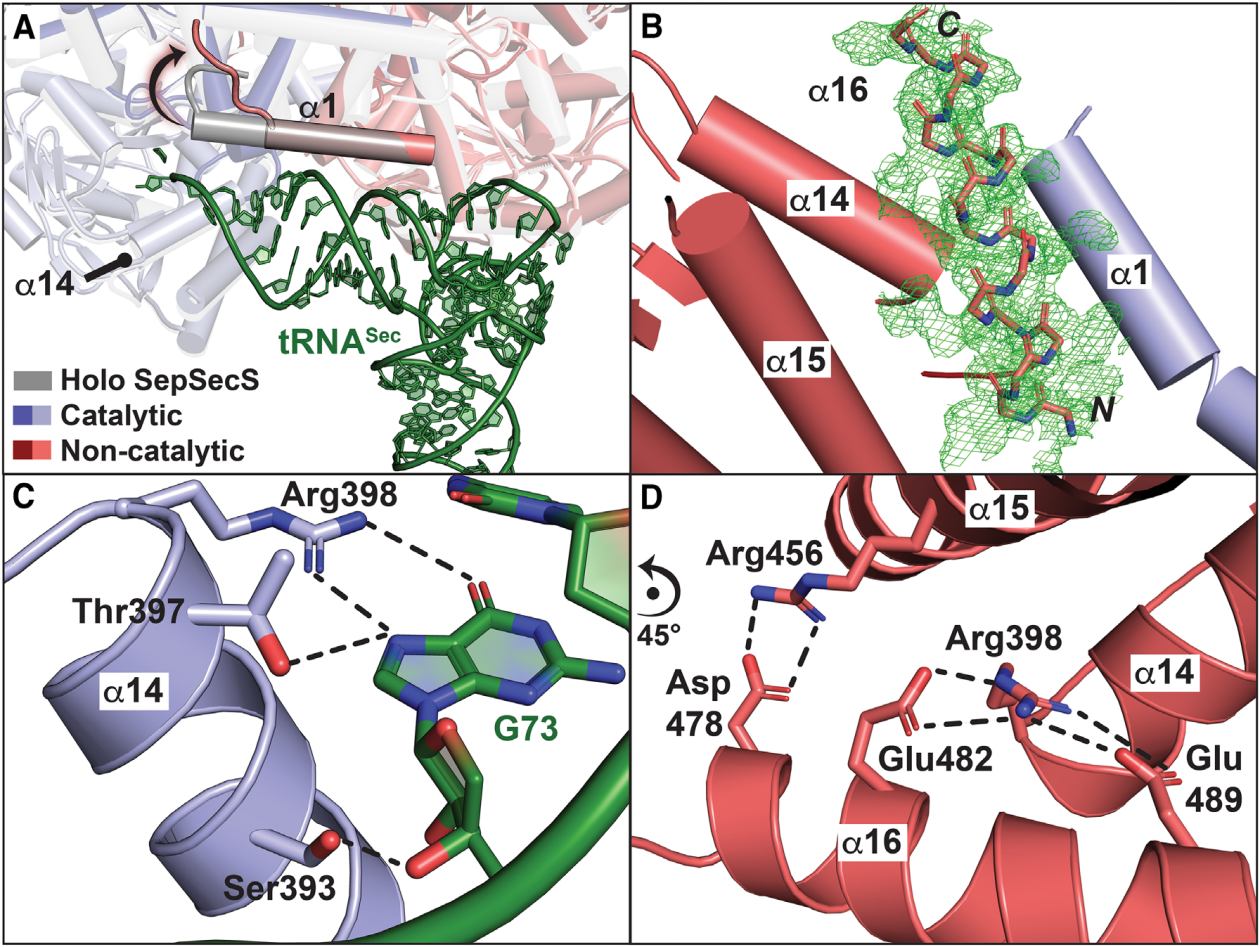
tRNA-induced conformational changes in SepSecS. (**A**) Overlay of holo- (grey cartoon) and tRNA-bound SepSecS (blue- and red-shaded cartoon) revealed that tRNA^Sec^ (green cartoon) binding alters the extreme N-terminus of α1 of each monomer of the docking SepSecS protomer into a coiled conformation to accommodate tRNA^Sec^. (**B**) Feature-enhanced electron density maps (green mesh; contour level 1.0σ) revealed a newly observed, C-terminal helix, α16 (pink poly-Ala sticks). The α16 helices from the non-catalytic protomer insert and stabilize between α14 of the same (pink cartoon) and α1 of the opposite (blue cartoon) protomer. (**C**) In the catalytic protomer, with accessible active sites, a highly conserved Arg398 of α14 establishes tRNA^Sec^ (green cartoon) identity through H-bonds with the Hoogsteen face of the discriminator base G73. (**D**) By contrast, in the non-catalytic protomer, α16 helices interact with α14 and are juxtaposed to the neighboring α1, thereby occluding the active sites of the non-catalytic protomer and blocking additional tRNA-binding events.

Our results showed that tRNA^Sec^ binding induces both short- and long-range restructuring of the extreme termini of the non-catalytic protomer. In the new complex structures, we could trace the protein backbone out to Arg11, thus adding seven residues to the previously visualized protein register. Importantly, in the non-catalytic protomer, a turn of N-terminal α1 (residues 18–20) unwinds and the segment encompassing residues 11–20 assumes a coiled conformation (Figure 2A). The extreme N-terminus folds upwards and away from the active site entrance. Given its proximity to the CCA-end of the bound tRNA^Sec^, the structural adjustment and movement of the extreme N-terminus may help the aminoacylated tRNA^Sec^ substrate access the active site of the neighboring catalytic protomer.

tRNA binding also reshapes the extreme C-terminus of the non-catalytic protomer. The more detailed mF_o_-DF_c_ electron density difference maps divulged an additional α-helix sandwiched between α14 of the non-catalytic protomer and α1 of the catalytic protomer (Figure 2B). While this helical density was also present in the SeMet-derivatized structure, the maps derived from the native complex crystals were of higher quality in this region. The lack of electron density for a linker between the new helix and the rest of the protein created an ambiguity as to whether the helix belonged to the N-terminus of the catalytic protomer or the C-terminus of the non-catalytic protomer. Moreover, secondary structure prediction algorithms suggested that SepSecS possesses additional α-helices at both the N- (residues 3–11) and C-termini (residues 481–491) (Supplementary Figure S3).

The new α-helix features side-chain densities reaching out towards Arg398 of the non-catalytic protomer, suggesting the new helix possesses acidic residues that engage in electrostatic interactions with α14 (Figure 2B). Importantly, in the catalytic protomer, Arg398 interacts with the Hoogsteen face of the G73 discriminator base to establish tRNA^Sec^ identity (Figure 2C). Given that the extreme C-terminus of SepSecS is markedly acidic, we modeled residues from Glu477 to Leu493 as helix α16 (Supplementary Figure S4). The resulting register positions Glu482 and Asp489 within H-bonding distance from the guanidium group of Arg398 (Figure 2D). These close contacts with α16 prevent Arg398 of the non-catalytic protomer from engaging with G73 of tRNA^Sec^ as the analogous Arg398 residues from the catalytic protomer do (Figure 2D). The rest of α16 sterically blocks the active site in the non-catalytic monomers, thereby precluding the non-catalytic protomer from catalyzing the reaction (Figure 2B). Interestingly, the overall occupancy of α16 was 100%, whereas tRNA occupancy in each binding site was approximately 50%. Thus, the crystal structure suggested that a single tRNA binding event alters the conformation of the extreme C-termini in two monomers, breaking the equivalency of the tRNA-binding sites in human SepSecS. In other words, docking of the first tRNA induces conformational changes that define the catalytic and non-catalytic nature of the SepSecS protomers.

Altogether, our results demonstrated that tRNA-induced conformational changes in the N- and C-termini of SepSecS lead to the structural asymmetry of the SepSecS•tRNA^Sec^ complex (43), which may be functionally relevant.

### tRNA^Sec^ and anions stabilize the active site conformation in the catalytic SepSecS protomer

The similarly modeled P-loop (residues Gly96–Lys107) in the active sites of all previous structures (32,41,43) implied such a pre-ordered P-loop conformation was catalytically competent. Phosphate and sulfate anions stabilized the P-loop in murine and archaeal holo SepSecS, respectively (33,41), while phosphoserine and thiophosphate stabilized the same conformation in the initial human SepSecS•tRNA^Sec^ crystal structure (32). However, with a minimally altered P-loop and no obvious structural changes in the active site, it was unclear how the SepSecS catalytic cycle would proceed. Our new structures demonstrated that both tRNA^Sec^ and small ligands induce structural rearrangements in the P-loop that may organize the SepSecS active site into a catalytically competent state.

**Figure 3.**
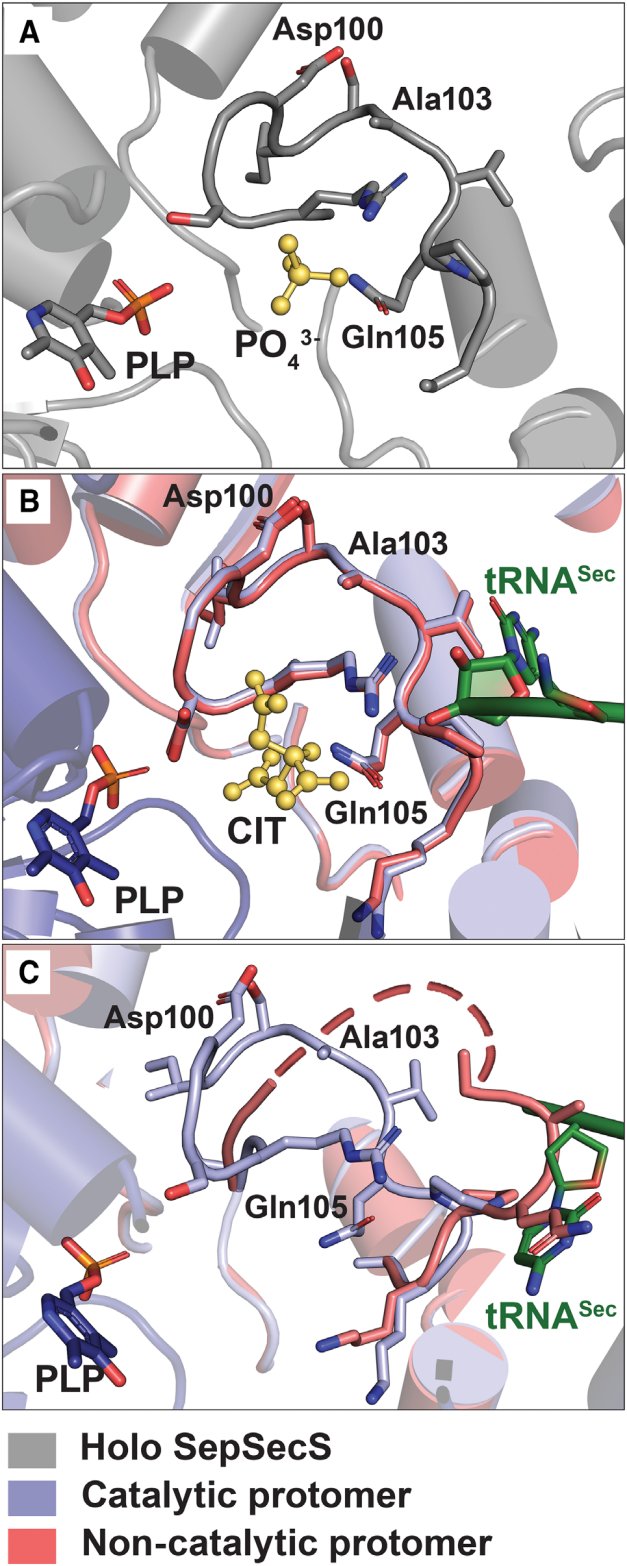
Substrate binding stabilizes the P-loop conformation in SepSecS. (**A**) Binding of a phosphate ion (PO_4_^3−^; yellow ball-and-stick) stabilizes the P-loop and active site in holo SepSecS (grey cartoon). (**B**) Citrate anions (CIT; yellow ball-and-stick) and tRNA^Sec^ stabilize the active sites of both protomers in the native SepSecS•tRNA^Sec^ complex. (**C**) Without a substrate or ligand, the active site and P-loop of the non-catalytic promoter (pink cartoon) remains largely disordered (pink dashed line) in the SeMet-SepSecS•tRNA^Sec^ complex. Conversely, binding of tRNA^Sec^ (green ball-and-stick) is sufficient to organize the active site of the catalytic promoter (light blue cartoon). In all panels, the Lys284-PLP covalent linkage has been omitted for clarity.

Our 2.25-Å resolution structure of holo SepSecS possessed a phosphate ion bound to the P-loop (Figure 3A) in a distinct binding pocket as previously observed (33,41). Additionally, our new crystal structure of the native SepSecS•tRNA^Sec^ complex, obtained under high-citrate concentrations, revealed that citrate bound to a similar site near the P-loop in both the catalytic and non-catalytic protomers (Figures 3B). An intriguing prospect of citrate binding is that cellular citrate or similar metabolites may regulate Sec synthesis. The overall positive electrostatic potential of the catalytic groove of SepSecS accommodates large anions mimicking selenophosphate, thiophosphate, or the sugar-phosphate backbone of the tRNA (Supplementary Figure S5). By contrast, the isomorphous SeMet-SepSecS•tRNA^Sec^ complex structure, obtained under low-citrate concentrations, harbored active sites devoid of large anions. Remarkably, while the P-loops are ordered in the catalytic protomer, they are disordered in the non-catalytic protomer (Figure 3C), presumably adopting two or more conformations. Moreover, in the absence of tRNA^Sec^ or large anions, the predominant conformation of P-loop residues (Ala103-Lys107) clashes with the placement of tRNA^Sec^ in the catalytic protomer. Thus, positioning of tRNA^Sec^ into the active site requires organization of the P-loop.

Based on our structural data, we propose that tRNA^Sec^ binding is a pre-requisite for ordering the P-loop into a catalytically competent state that accommodates entry of the CCA-end into the active site, while small ligand binding may additionally stabilize the active sites in SepSecS. Together the tRNA and ligand binding pockets could help the enzyme distinguish different steps in the reaction cycle.

### Polar interactions govern binding of SepSecS to tRNA^Sec^ in solution

**Figure 4.**
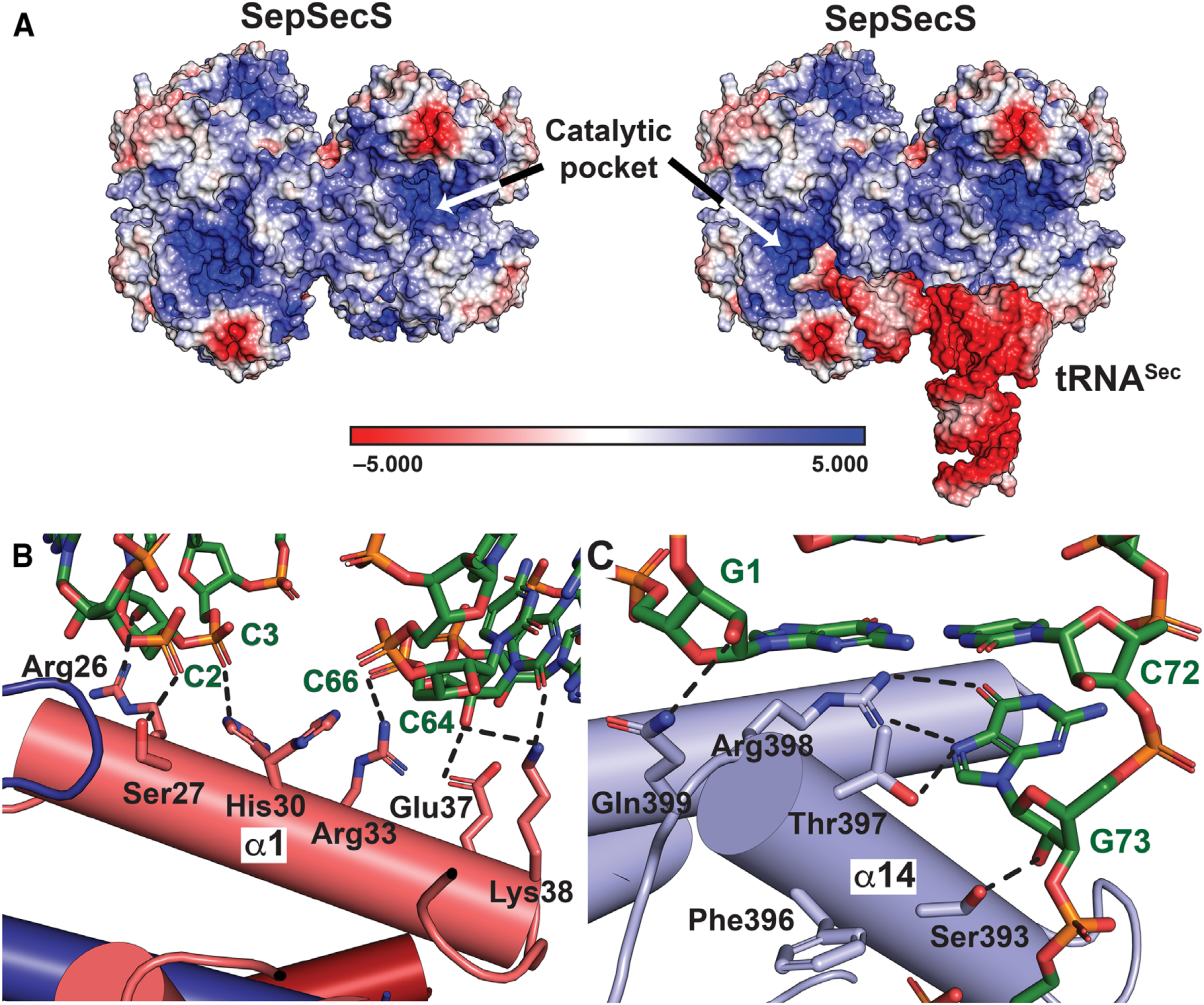
Electrostatic interactions govern complex formation between SepSecS and tRNA^Sec^. (**A**) Electrostatic potential maps of the holo (left) and tRNA^Sec^ docked onto holo SepSecS (right) showed that positively charged surfaces (blue) that constitute the tRNA^Sec^ binding pocket of SepSecS are complementary to the negatively charged sugar-phosphate backbone of tRNA^Sec^ (red). The active site of SepSecS also exhibited strong positive potential. As illustrated by the bar, surfaces are contoured from –5 (red) to +5 (blue) kT/e^−^ based on the potential of the solvent accessible surface. Hydrogen bonds (black-dashed lines) mediate interactions between tRNA^Sec^ and α1 (**B**) and α14 (**C**).

Mapping the electrostatic potential onto the surfaces of SepSecS and tRNA^Sec^ illustrated that positively charged catalytic pockets in SepSecS are complementary to the negative charges on the tRNA^Sec^ backbone and phosphoserine and selenophosphate ligands (Figure 4A). Further examination of these surfaces in our crystal structures revealed that solvent-exposed residues in helices α1 and α14 of SepSecS and the sugar-phosphate backbone of the acceptor and TΨC arms of tRNA^Sec^ comprise the complementary electrostatic surfaces (Figures 4B, C).

Surprisingly, sequence conservation of polar residues of α1 is weak (Supplementary Figure S3). This lack of conservation implies that the presence of hydrophilic amino acids, and not their identity, is sufficient to engage with tRNA^Sec^. Conversely, hydrophobic residues of α1 are conserved as they anchor α1 within the α1-α2-α1-α2 tetramerization motif. Consistent with their direct role in recognizing and orienting ^73^GCCA^76^ of tRNA^Sec^, stronger sequence conservation is present in α14 (Supplementary Figure S3), especially in positions 396–398 (Figure 4C). To corroborate the significance of electrostatic interactions in mediating the SepSecS:tRNA^Sec^ interaction, we performed MST-based assays to determine the dissociation constant (*K*_d_) and thermodynamic parameters (i.e. Δ*H*° and Δ*S*°) of complex formation.

**Figure 5.**
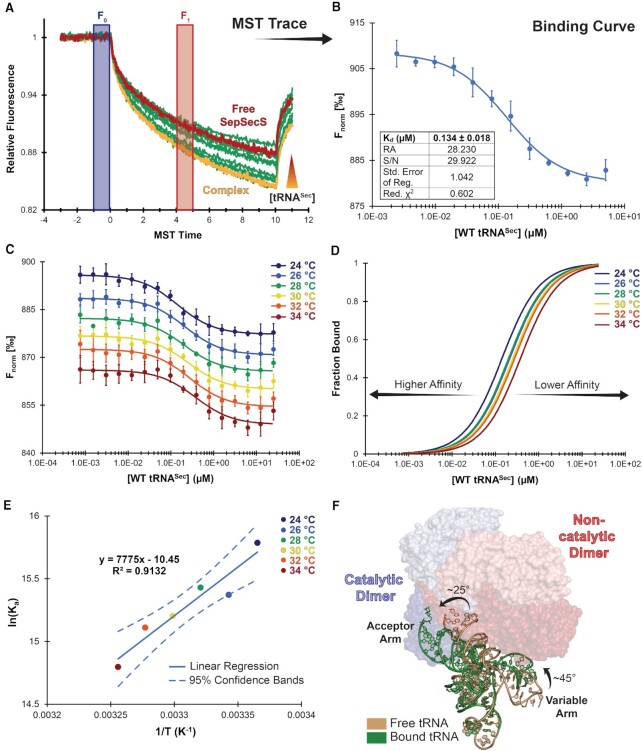
The SepSecS•tRNA^Sec^ complex formation is enthalpically driven. To determine the *K*_d_ of the SepSecS:tRNA^Sec^ interaction, the normalized fluorescence (F_norm_ in [‰]) of the labeled protein (**A**) was plotted against the concentration of WT tRNA^Sec^ (**B**). (**C**) The MST-generated binding curve for WT SepSecS binding to tRNA^Sec^ measured from +24–34°C. (**D**) Increasing the temperature lowered the binding affinity, requiring more substrate to reach saturation. (**E**) The van’t Hoff plot shows that complex formation is enthalpically favored and entropically unfavorable. (**F**) Superimposition of the crystal structures of free (PDBID 3A3A; dark yellow cartoon) and SepSecS-bound tRNA^Sec^ (PDBID 7MDL; green cartoon). Upon protein binding, the acceptor-TΨC helix of tRNA^Sec^ rotates 24° and translates 10 Å, while the variable arm rotates 43° and translates 11 Å. RA, response amplitude; S/N, signal-to-noise; Std. Error of Reg., standard error of regression; Red. *χ^2^*, reduced *χ*^2^.

We determined that WT SepSecS binds unacylated tRNA^Sec^ with *K*_d_ of 134 nM (Figure 5A–B), which is in good agreement with the *K*_d_ of 78 nM obtained using tryptophan fluorescence quenching (43). To calculate ΔH° and ΔS°, we performed MST experiments at +2 °C-intervals over a temperature range of +24 to +34°C (Figure 5C). As the temperature increased the binding affinity decreased, indicating that complex formation is an exothermic process (Figure 5D), as expected for an interaction mediated by electrostatics. The MST-derived van’t Hoff plot (*R*^2^ = 0.9132) yielded a Δ*H*° of −64.65 ± 11.30 kJ/mol and Δ*S*° of −0.0869 ±0.0374 kJ/(mol•K) (Figure 5E). These data characterize the SepSecS:tRNA^Sec^ interaction as an enthalpically driven and entropically restricted process, whereby electrostatic interactions drive complex formation. A pairwise alignment using the anticodon stem of SepSecS-bound and free tRNA^Sec^ illustrates the entropic cost of binding, as SepSecS induces strain in the acceptor, TΨC, and variable arms of tRNA^Sec^ (Figure 5F).

Thus, the thermodynamic data support a model in which a favorable enthalpy, derived from electrostatic interactions between the enzyme and tRNA, drives complex formation to overcome the cost of conformational stabilization.

### Probing the role of helices α1 and α14 of SepSecS in tRNA^Sec^ binding

After establishing that polar interactions mediate SepSecS•tRNA^Sec^ complex formation, we sought to investigate the contributions of individual residues in helices α1 and α14 in tRNA binding. Consequently, we engineered a series of enzyme mutants (e.g. R26A, S27A, H30A, E37L, K38M, F396V, T397V, R398A, R398E and Q399A) and evaluated their binding to tRNA^Sec^ using MST. Our results showed that primarily positive and solvent-exposed side chains in these helices are important for tRNA^Sec^ binding.

We first probed the structural integrity of mutant enzymes by monitoring their thermal unfolding profiles. The similar initial ratios and Δratios of the SepSecS mutants indicated the mutants have a similar aggregation status, while their comparable inflection temperatures (*T*_i_) suggest they follow the same unfolding trajectory and adopt the same structure as the WT enzyme (Supplementary Table S2). Subsequent MST analyses of α1 and α14 mutants provided a nuanced view on the role of individual side chains in tRNA^Sec^ binding and recognition. For instance, R26A and K38M caused an increase in the *K*_d_, whereas R33A marginally increased the affinity (Table 1, Supplementary Figures S6 and S7). S27A, H30A, and E37L recapitulated the WT K_d_ value, suggesting their negligible role in tRNA binding. In the case of the α14 mutants, we observed a similar range of effects. Perhaps the most striking result was from probing the functionally relevant and highly conserved Arg398, which forms H-bonds with the Hoogsteen face of the G73 discriminator base. Its replacement with Ala (R398A) weakened affinity by more than an order of magnitude, while substitution with Glu (R398E) abolished binding (Table 1, Supplementary Figure S8E). The Q399A mutant, which coordinates the 5′-phosphate binding pocket, slightly diminished the affinity, whereas S393A resembled the WT enzyme (Table 1, Supplementary Figures S6 and S8). Surprisingly, F396V and T397V were stronger tRNA^Sec^ binders, just like R33A (Table 1, Supplementary Figures S6 and S8). Here, we speculated that the removal of a flexible side chain (e.g. Phe and Arg) would decrease the entropy, permitting a closer contact with tRNA^Sec^ to further stabilize electrostatic interactions that could then increase the enthalpy of binding. Indeed, these higher-affinity mutants all displayed a marked reduction in the entropy of binding (Table 2) and a greater enthalpy of binding when compared to WT SepSecS (Supplementary Figure S9 and S10).

**Table 1.** Binding affinities between WT and mutant SepSecS constructs and WT tRNA^Sec^

SepSecS	*K*_d_ (μM)^a^
**WT**	0.134 ± 0.018
***α1 mutants***	
R26A	0.516 ± 0.125
S27A	0.133 ± 0.014
H30A	0.166 ± 0.034
R33A	0.0289 ± 0.0070
E37L	0.166 ± 0.026
K38M	1.67 ± 0.60
***α14 mutants***	
S393A	0.197 ± 0.022
F396V	0.0441 ± 0.0063
T397V	0.0628 ± 0.0100
R398A	2.53 ± 1.12
R398E	No binding
Q399A	0.567 ± 0.168

^a^Uncertainties are 68% confidence intervals.

**Table 2.** Thermodynamic parameters for high-affinity SepSecS mutants

SepSecS	Δ*H*° (kJ/mol)^a^	Δ*S*° (kJ/(mol•K))^a^
WT	−64.65 ± 11.30	−0.0869 ± 0.0374
***α1 Mutants***		
R33A	−80.00 ± 7.59	−0.132 ± 0.025
***α14 Mutants***		
F396V	−149.0 ± -8.1	−0.363 ± 0.027
T397V	−71.48 ± 4.37	−0.107 ± 0.014

^a^Uncertainties are 68% confidence intervals.

Taken together, our MST data validate a model of complex formation whereby α1 provides electrostatic interactions to aid tRNA docking, while α14 supplies specific residues that establish tRNA identity. However, the data revealed nuances of how SepSecS refines the strength of the interaction. For example, SepSecS appears to use bulkier side chains to weaken the binding affinity. Maintaining the binding affinity within a certain range may be important for ensuring efficient turnover of the product to the eEFSec and the Sec translational machinery. While our crystal structures revealed that Ser27, His30, Glu37 and Ser393 may form hydrogen bonds with the tRNA backbone atoms, their substitution with Ala had minimal effect on binding affinity. Perhaps, these contacts act in synergy or solvent molecules and/or protein backbone atoms could replace these interactions with ease.

### Significance of α1 and α14 of SepSecS in selenoprotein synthesis

**Figure 6.**
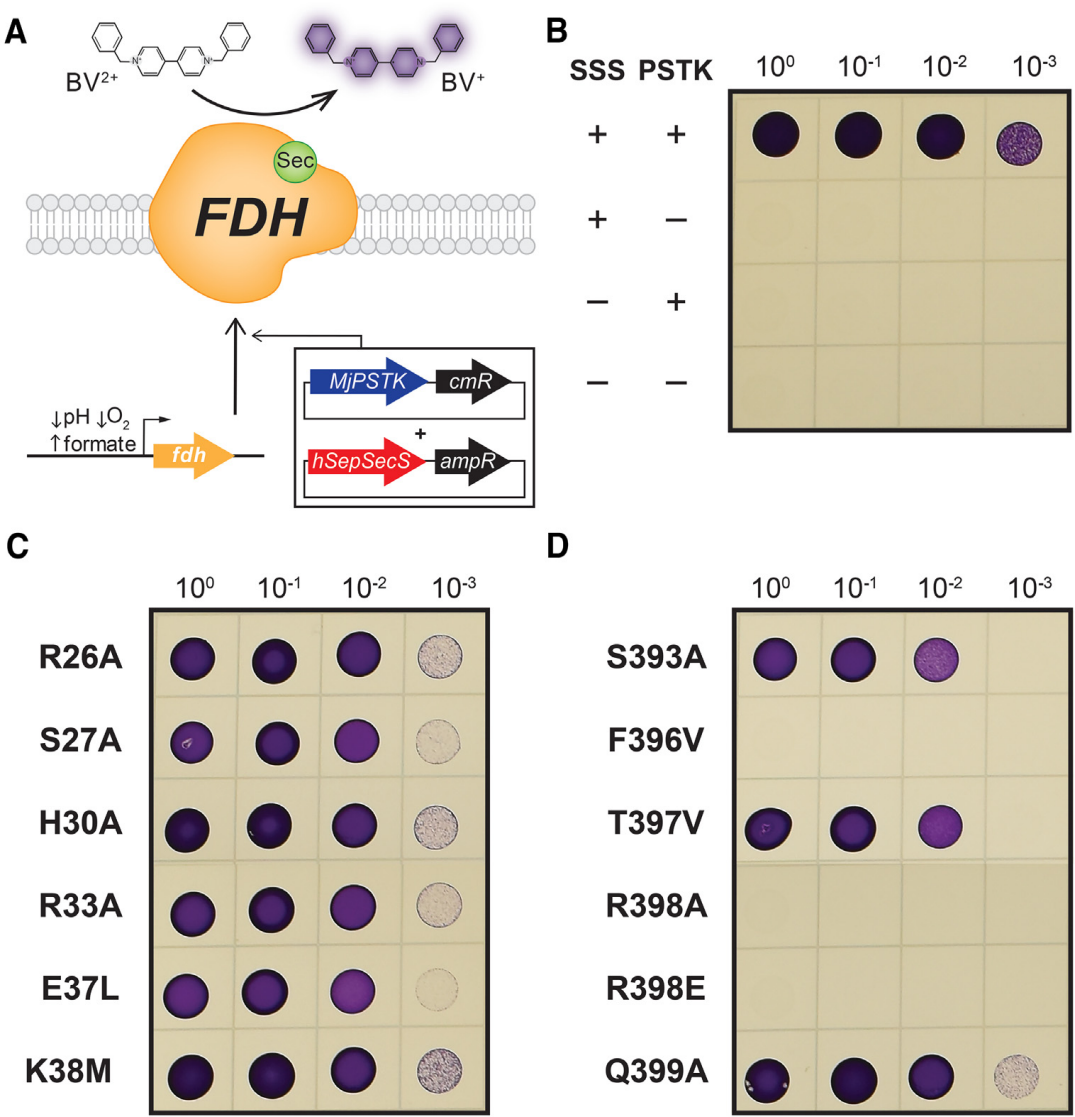
*E. coli*-based assay for evaluation of the catalytic activity of SepSecS mutants. (**A**) Co-expression of human SepSecS and *M. jannaschii* PSTK (MjPSTK) allows Δ*selA* JS2(DE3) cells to synthesize formate dehydrogenase (FDH) and reduce benzyl viologen (BV). (**B**) Only co-expression of SepSecS and PSTK compensates for Δ*selA* and restores FDH synthesis and activity throughout the dilution series, leading to BV reduction and purple bacterial colonies. (**C**) Mutants mapping to α1 exhibit similar activity as the WT. (**D**) α14 mutants F396V, R398A, and R398E lost catalytic activity; S393A and T397V are active at a reduced level, while the activity of Q399A is the same as that of the WT enzyme.

MST assays characterized the binding of SepSecS mutants to unacylated tRNA^Sec^ but were uninformative regarding their contribution to catalysis. Thus, we delineated whether any of the residues in helices α1 and α14 play a functional role during selenoprotein synthesis using a well-established benzyl viologen (BV)-based *Escherichia coli* complementation assay (33). This indirect activity assay evaluated whether co-expression of SepSecS and archaeal PSTK could compensate for the loss of SelA to enable synthesis of a bacterial selenoenzyme, formate dehydrogenase (FDH) under anaerobic conditions in a Δ*selA* bacterial strain (Figure 6A). This system demonstrated that SepSecS catalytic competency is tolerant to mutations in α1, but especially sensitive to mutations in α14. Thus, the role of α14 residues in orienting the ^73^GCCA^76^ end is essential to the enzyme achieving catalytic efficiency.

Only co-expression of catalytically active SepSecS and PSTK rescued FDH expression, allowing reduction of the BV substrate from its colorless oxidized form to a reduced, purple form (Figure 6B). Interestingly, the co-expression conveyed a growth advantage, likely due to the ability of the host *E. coli* cells to metabolize formate as an energy source (Supplementary Figure S11). The advantage was evident by the denser growth of *E. coli* cells on agar plates. Further, though displaying a range of *K*_d_ values for tRNA^Sec^ binding (29 nM–1.7 μM), α1 mutants of SepSecS reduced BV equally well as the WT enzyme, arguing that SepSecS can form a productive complex with tRNA^Sec^ over a wide range of binding affinities (Figure 6C). Conversely, apart from Q399A, mutations affecting solvent-exposed residues in α14 largely led to catalytic impairment. Residues S393A and T397V exhibited a minor deficiency in catalysis at the lowest dilution level, whereas R398A and R398E were completely inactive over the entire dilution range, consistent with earlier functional results (32). Given that R398E was unable to bind tRNA^Sec^, the lack of catalysis was expected (Table 1). Surprisingly, the high affinity F396V mutant was also incapable of catalysis. Western blots confirmed that all strains expressed PSTK and either WT or a mutant SepSecS (Supplementary Figure S12), thus the absence of BV reduction was solely due to a loss of function and not lack of expression.

### The acceptor-TΨC arm of tRNA^Sec^ is the major recognition determinant for SepSecS

Because human SepSecS binds primarily to the acceptor-TΨC and variable arms, we speculated that these two elements (Supplementary Figure S13A) may be the major recognition motifs in tRNA^Sec^ (32). To assess their significance for complex formation, we employed mutational studies and MST binding assays. We engineered bacterial-like 8/5-fold (Mut 3) and canonical-like 7/5-fold (Mut 5) tRNA^Sec^ mutants (Supplementary Figure S13B, versus C) (26), as well as hybrid constructs which either completely lacked the variable arm (ΔVar) or harbored the extended variable arm from tRNA^Ser^ (vSer) (Supplementary Figure S13D).

Our MST data established that Mut3, ΔVar, and vSer bind to WT SepSecS (Table 3 and Supplementary Figure S14). Given its promiscuity towards bacterial tRNA^Sec^, the binding of SepSecS to the 8/5-fold Mut3 was expected. However, the binding affinity was significantly lower (∼2 μM versus 134 nM) compared to WT tRNA^Sec^. Conversely, the SepSecS interaction with Mut5 tRNA^Sec^ exhibited a binding curve with the right part of the curve trailing up with no plateau, indicating that SepSecS cannot specifically engage with Mut5 (Supplementary Figure S14D). Consequently, we concluded that the 13 bp-long acceptor-TΨC arm of tRNA^Sec^ is the major determinant for SepSecS recognition. On the other hand, the extended variable arm of vSer raised the *K*_d_ to 449 nM, while binding to the ΔVar mutant that lacked a variable arm resembled the *K*_d_ value for WT tRNA^Sec^. Taken together, the variable arm of tRNA^Sec^ does not appear to be a recognition element for SepSecS but may help the enzyme discriminate against tRNAs with extended variable arms, such as tRNA^Ser^.

**Table 3.** Binding affinities between WT SepSecS and tRNA^Sec^ mutants

tRNA^Sec^ construct	*K*_d_ (μM)^a^
WT	0.134 ± 0.018
***Variable arm mutants***	
vSer	0.449 ± 0.146
ΔVar	0.171 ± 0.061
***Acceptor-T* *Ψ* *C arm mutants***	
Mut3 (8/5)	2.04 ± 1.07
Mut5 (7/5)	Non-specific binding

^a^Uncertainties are 68% confidence intervals.

## DISCUSSION

Recognition of a specialized tRNA^Sec^ and its discrimination from canonical tRNAs was crucial for the expansion of the genetic code to incorporate Sec into selenoproteins while maintaining translation fidelity (56). tRNA^Sec^ possesses extended acceptor, TΨC, D- and variable arms compared to canonical tRNAs that aid the Sec synthetic machinery in their recognition and discrimination of tRNA^Sec^. Here, we sought to delineate the precise elements governing formation of a productive complex between human SepSecS and tRNA^Sec^.

Previous studies proposed that human SepSecS adopts a pre-ordered conformation for a high-affinity interaction with tRNA^Sec^ (32,41,43). Substrate binding was believed to occur by a sequential mode of allosteric regulation, where binding of one tRNA^Sec^ molecule facilitated binding of second tRNA^Sec^ to the cross-dimer and reduced the binding affinities of the non-catalytic protomer (43). Within this model, it remained unclear how a pre-ordered enzyme could perceive substrate acquisition or product release and what could be the mechanism for allosteric regulation (40,57). Recently, we obtained new high-resolution crystal structures which revealed novel features of SepSecS that hinted at an alternative mechanism of complex formation. To resolve these questions within the framework of our new structures, we deployed a combination of biochemical, biophysical, and functional assays.

Electrostatic potential mapping indicated that SepSecS employs charge-based interactions to recognize and engage tRNA^Sec^. MST data confirmed that the enzyme uses α1 and α14 residues to engage in polar interactions with tRNA^Sec^ to generate a favorable binding enthalpy that compensates for the entropic cost of stabilizing the tRNA^Sec^ conformation and elements in SepSecS. Our measurements affirmed that SepSecS primarily recognizes the extended 13-bp long acceptor-TΨC fold of tRNA^Sec^. On the other hand, the variable arm of tRNA^Sec^ may serve as a discriminatory element. While most tRNAs have four or five nucleotides in their variable loop, class II tRNAs (including tRNA^Sec^) have 10 or more nucleotides (58). Our MST data suggests that SepSecS may discriminate against other class II tRNAs with extended variable arms, such as tRNA^Ser^. Additionally, this element may serve as an anti-determinant preventing false recognition by aminoacyl-tRNA synthetases other than SerRS and other enzymes and factors involved in protein translation (37). This quality check could help prevent mis-incorporation of Ser and phospho-Ser, but not Cys, at Sec UGA codons.

Surprisingly, α1 residues that contribute to tRNA binding minimally impact catalysis, as the enzyme could sustain catalysis over a wide range of binding affinities (K_d_ from 29 nM-1.7 μM). By contrast, nearly all α14 mutants exhibited impaired catalysis. This impairment concurs with structural data showing that conserved α14 residues deliberately engage the ^73^GCCA^76^-3′ end of tRNA^Sec^ (Figure 4C). Thus, α14 residues do not merely aid substrate binding, but actively participate in orienting and positioning the CCA-end and the attached phosphoseryl moiety within the active-site groove for catalysis. Since Arg398 directly engages with the G73 discriminator base, its import is clear. However, the role of Phe396 was not as unambiguous. We had previously speculated that Phe396 forms π-stacking interactions with one of the nucleobases of the CCA-end. However, the CCA-end was poorly resolved in our crystal structures, and the F396V mutation minimally strengthened binding affinity, indicating that Phe396 is not essential to the binding energy. Our MST data also demonstrated that F396V caused a significant loss of entropy, implying that the F396V complex adopts a productive-like conformation but with a more rigid CCA-end. Such rigidity could impair optimal positioning of the phosphoseryl moiety near the P-loop and PLP or hinder movement of the aminoacyl group through the catalytic cycle. Likewise, introduction of Val in place of Thr397, which interacts with N7 of the G73 discriminator just upstream of the CCA-end, decreased the entropy of binding and impaired catalysis. Altogether, our results argue that the CCA-end requires some flexibility for optimal catalysis. Conservation of an aromatic residue in the Phe396 position in archaea and eukaryotes, and conservation of Thr397 among vertebrates supports proposed roles for these residues in catalysis (Supplementary Figure S3).

The apparent contradiction that α1 residues that participate in tRNA binding negligibly affect catalysis could be because the functional assay relied on the interaction between human SepSecS and bacterial SelC and not human tRNA^Sec^. Given the strict conservation of ^73^GCCA^76^-3′-end across all tRNA^Sec^ species, the interaction of α14 with either SelC or tRNA^Sec^ should be similar (39), but significant differences may be present at the interface between α1 and the acceptor arm. The difficulties in synthesizing large quantities of Sep-tRNA^Sec^ (55,59) limited our structural and *in vitro* experiments to using unacylated tRNA^Sec^. Thus, we could not interrogate the role of the aminoacyl moiety in the binding and catalysis by SepSecS. Hence, enzymatic studies that could directly determine *k*_cat_ and *K*_M_ values of the SepSecS-catalyzed reaction would further elucidate the mechanisms of enzyme turnover and catalysis. Alternatively, the similar catalytic efficiency of SepSecS over a wide range of tRNA^Sec^ binding affinities (Figure 6C, D) may instead reflect that SepSecS and tRNA^Sec^ are components of a multi-enzyme Sec-synthetic complex in the cell (60,61). Such a complex would improve the efficiency of Sec synthesis and limit Se toxicity, while being compatible with the half-sites occupancy of SepSecS. Within such a larger complex, a single mutation in α1 could have minimal impact on tRNA^Sec^ binding and catalysis, as we observed in our study.

**Figure 7.**
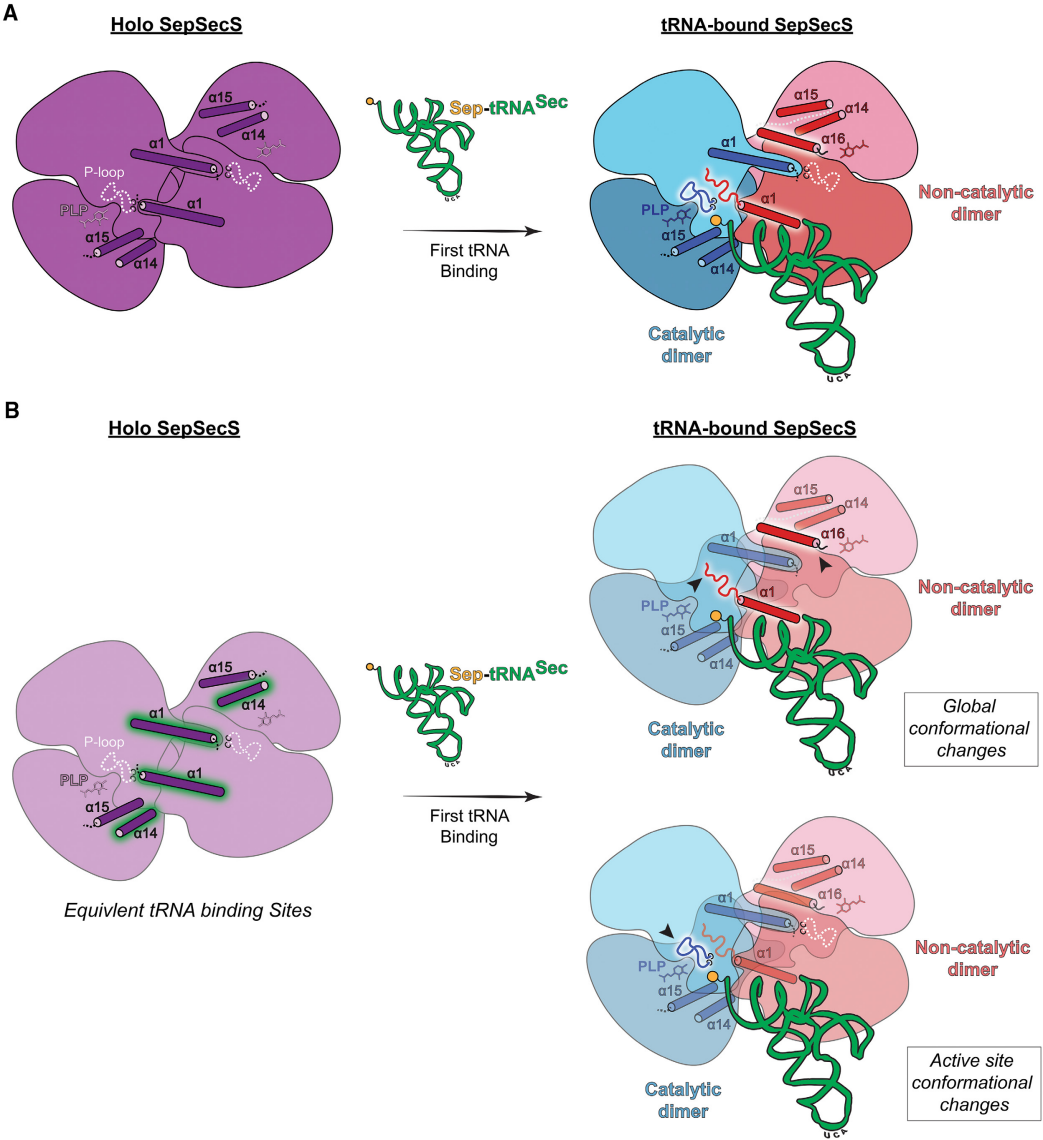
The revised model of SepSecS activation. (**A**) tRNA^Sec^ binding induces a conformational change in SepSecS to achieve a catalytically competent organization. (**B**) Breakdown of steps characterizing the conformational rearrangement. Tetrameric holo SepSecS possess four equivalent substrate binding sites (highlighted helices). Binding of one tRNA molecule induces a global rearrangement of the enzyme into a catalytic and non-catalytic protomers. α1 shifts up (left arrowhead, top) and uncoils to accommodate the substrate. At the same time α16 docks into the tRNA binding pockets of the cross dimer (right arrowhead, top), precluding substrate binding and defines this unit as the non-catalytic promoter. Within the active site, phosphoseryl-tRNA^Sec^ (Sep-tRNA^Sec^) stabilizes the active site P-loop in preparation for catalysis (arrowhead, bottom). Subsequently, only a second pocket on the opposite face of the enzyme is available for binding and catalysis.

Altogether, binding and functional studies combined with our high-resolution crystal structures of holo and tRNA-bound SepSecS delineate a revised model of the terminal Sec-synthetic reaction (Supplementary Video S1). In holo SepSecS, all four monomers are equivalent, possessing disordered catalytic P-loops and C-termini (Figure 7A). Upon binding of the first Sep-tRNA^Sec^, the N-terminus of the docking SepSecS monomer extends, unwinds into a coiled conformation, and tilts away from the active site entrance to accommodate the acceptor arm of tRNA^Sec^ and allow access of its CCA-end to the active site. Binding stabilizes α16 in the neighboring monomers, causing steric occlusion of their tRNA-binding and catalytic sites, thus breaking the equivalency of these sites within the tetramer (Figure 7B, top). Since the N-terminus participates in the tetrameric interface, tRNA-induced changes in this region could relay substrate binding across the enzyme, such that tRNA^Sec^ binding in one monomer promotes stabilization of α16 in the two neighboring monomers (31). These conformational changes lead to a clear demarcation of the ‘docking’, non-catalytic and catalytic SepSecS protomers. Binding of substrates, large anions and/or tRNA^Sec^, is sufficient to organize the P-loop of the enzyme (Figure 7B, bottom), perhaps via a mechanism of induced fit or conformational selection. Given that the anions may mimic the selenophosphate donor and phosphate leaving group (32), their binding may inform the enzyme about its state along the reaction coordinate. In the end, our results show that tRNA^Sec^ binding initiates a series of conformational adjustments that facilitate transition of the holoenzyme into a catalytically competent state. However, additional studies addressing the physiological relevance of the half-sites occupancy of SepSecS are warranted.

Our study provides a foundation for further manipulation of the SepSecS•tRNA^Sec^ interaction to address unanswered questions about the Sec translational machinery and selenoprotein synthesis. Moreover, because the catalytic mechanism of SepSecS involves the anhydroalanyl species, modulation of this enzyme could lead to engineering of a direct system for synthesis of covalently modified proteins, which would be of immense value in the realm of synthetic biology.

## DATA AVAILABILITY

The coordinates and structure factors are deposited in PDB with the accession codes 7L1T (for holo SepSecS), 7MDL (for SepSecS•tRNA^Sec^) and 8G9Z (for SeMet-SepSecS•tRNA^Sec^).

## Supplementary Material

gkad182_Supplemental_Files

## Notes

Present address: Jennifer Castillo Suchkou, Rush Medical College, Rush University Medical Center, Chicago, IL 60612, USA.

Present address: Rachel L. French, Agilent Technologies Inc., Santa Clara, CA, USA.

Present address: Miljan Simonović, National Institutes of Health, National Institute of General Medical Sciences, Bethesda, MD 20892, USA.
