# Supplementary material for: Structural basis for the tRNA-dependent activation of the terminal complex of selenocysteine synthesis in humans
Source: Nucleic Acids Res. 2023 Mar 17;51(8):4012–26. doi: 10.1093/nar/gkad182 (PMC10164584; doi:10.1093/nar/gkad182)
Supplement: gkad182_Supplemental_Files [file gkad182_supplemental_files.zip › Supplemental Video 1 Legend.docx]

**Supplemental Video 1. Revised model of substrate-induced activation of human SepSecS.** Holo-SepSecS is a homotetramer possessing four equivalent tRNA^Sec^ binding sites (highlighted helices) comprised in part by α14 from one monomer and α1 from a neighboring monomer. In the absence of substrate, the catalytic P-loop of each monomer is disordered. Binding of a single tRNA molecule induces a global rearrangement of the enzyme into a catalytic and non-catalytic protomers. α1 shifts up and uncoils to accommodate the substrate. At the same time α16 helices dock into the tRNA binding pockets of the cross dimer, precluding substrate binding, which defines this unit as the non-catalytic promoter. Within the active site, phosphoseryl-tRNA^Sec^ (Sep-tRNA^Sec^) stabilizes the active site P-loop in preparation for catalysis. Consequently, on opposing face of the tetramer, only one of the two tRNA binding sites remains open to bind a second tRNA molecule. Binding of Sep-tRNA^Sec^ to the remaining site then stabilizes a second active site P-loop in preparation for catalysis.
