# Supplementary material for: Structural basis for the tRNA-dependent activation of the terminal complex of selenocysteine synthesis in humans
Source: Nucleic Acids Res. 2023 Mar 17;51(8):4012–26. doi: 10.1093/nar/gkad182 (PMC10164584; doi:10.1093/nar/gkad182)
Supplement: gkad182_Supplemental_Files [file gkad182_supplemental_files.zip › SOM_Rev.docx]

**Supporting Online Materials**

**Structural basis for the tRNA-dependent activation of the terminal complex of selenocysteine synthesis in humans**

Anupama K. Puppala *, Jennifer Castillo Suchkou^$^, Rachel L. French ^#^, Kaitlyn A. Kiernan, Miljan Simonović ^&,^*

Department of Biochemistry and Molecular Genetics, University of Illinois at Chicago, Chicago, Illinois 60607, USA

* Corresponding authors:

Miljan Simonović, PhD

National Institutes of Health

National Institute of General Medical Sciences

Bethesda, MD 20892

USA

Email: [miljan.simonovic@nih.gov](mailto:miljan.simonovic@nih.gov)

Anupama Puppala, PhD

Department of Biochemistry and Molecular Genetics

University of Illinois at Chicago

900 S. Ashland Ave., 1354 MBRB

Chicago, IL 60607

U.S.A.

Email: [puppala2@uic.edu](mailto:puppala2@uic.edu)

^$^ Present address: Rush Medical College, Rush University Medical Center, Chicago, IL 60612, USA

^#^ Present Address: Agilent Technologies Inc., Santa Clara, CA, USA

^&^ Present address: National Institutes of Health, National Institute of General Medical Sciences, Bethesda, MD 20892, USA

**SUPPLEMENTAL MATERIAL AND METHODS**

**Cloning of SepSecS, PSTK, and tRNA^Sec^**. Human SepSecS and tRNA^Sec^ were previously cloned into pET15b and pUC19 vectors (1). Mutant constructs of SepSecS and tRNA^Sec^ were generated using mutagenic primers and the QuikChange II XL Site-Directed Mutagenesis Kit (Agilent). Sanger sequencing confirmed the presence of the desired mutations.

For the SepSecS *in vivo* assay, a codon-optimized PSTK from *Methanocaldococcus jannaschii* (MjPSTK), possessing a N-terminal Myc tag was placed into a pACYCDuet-1 vector using NdeI and XhoI restriction sites (Genscript). Either the pACYCDuet-1 or pACYCDuet-1-PSTK were transformed into the Δ*selA* JS2(DE3) strain. After selecting a single colony for each strain, electrocompetent cells were prepared. Subsequently, either the pET15b empty vector, pET15b-WT SepSecS, or pET15b-mutant SepSecS were transformed into these JS2(DE3) cells to generate strains possessing dual pET and pACYC vectors.

**Expression and purification of SepSecS.** For large-scale expression of SepSecS, the pET15b vector carrying either the WT or a mutant SepSecS was transformed into C41(DE3) cells and plated onto LB agar plates with ampicillin (100 μg/mL). A single colony was selected and grown to density overnight in LB. This starter culture was used to inoculate 4 L of LB media with ampicillin (100 μg/mL) at a ratio of 1:100. The 4 L culture grew at +37 °C until the cells reached log phase, at an optical density of 0.6-0.8 at 600 nm (OD_600_). To induce protein expression, IPTG was added to a final concentration of 0.5 mM. The cultures then continued to grow at +15 °C for 18-20 h. The cells were harvested by spinning the cultures in a centrifuge at 5,000 rpm. The pelleted cells were flash-frozen using liquid nitrogen and stored at -80 °C.

The selenomethionine (SeMet)-derivatized human SepSecS (SeMet-SepSecS) was expressed by the metabolic inhibition method (2). Briefly, the cells were grown at +37 °C in M9 minimal media supplemented with ampicillin (100 μg/mL), 0.4% glucose, 2 mM MgSO_4_, 0.1 mM CaCl_2_, 1x vitamin B mixture, and 40 mg/L of all 19 amino acids excluding Met. When cultures reached an OD_600_ of 0.6, Met inhibition and SeMet incorporation was induced by adding 50 mg/L Thr, Phe, and Lys, 25 mg/L Leu, Ile, and Val, and 60 mg/L SeMet to the cultures, which were grown for an additional 20 min. Subsequently, protein expression was induced by addition of 0.5 mM IPTG, and the cultures grew at +15 °C for 18-20 h.

For SepSecS purification, the cells were lysed by sonication in lysis buffer (50 mM HEPES, pH 8.0, 300 mM NaCl, 10% (v/v) glycerol, 10 mM imidazole, 10 µM PLP and 3 mM βMe) supplemented with a protease inhibitor tablet (Roche), using 4 mL of lysis buffer per gram of cell pellet. To separate the soluble cell lysate from the cell debris, the sonicated suspension was centrifuged at 15,000 rpm for 45 min. The soluble lysate was then loaded onto an FF Crude Ni^2+^-affinity column (Cytiva) equilibrated with lysis buffer. The column with was rinsed with 200 mL of lysis buffer, followed by 200 mL of wash buffer (lysis buffer with 60 mM imidazole). Finally, affinity purified SepSecS was eluted with elution buffer (lysis buffer with 350 mM imidazole). The SepSecS eluate was loaded onto a S200 16/600 Superdex (Cytiva) size-exclusion chromatography (SEC) column that was pre-equilibrated with 20 mM HEPES, pH 8.0, 200 mM NaCl, 5% (v/v) glycerol, 10 µM PLP, and 0.5 mM TCEP. The purified sample was concentrated using a 30 kDa MWCO centrifugal concentrator (Amicon), filtered through a 0.22 µm filter, flash-frozen in liquid nitrogen, and stored at -80 °C. Concentration and purity of the purification process was assessed by SDS-PAGE. SeMet SepSecS was purified in the same manner except for a higher concentration of reducing agents in purification buffers (10 βME and 5 mM TCEP).

***In vitro* transcription and purification of tRNA^Sec^**. Human tRNA^Sec^ was synthesized using *in vitro* T7 RNA polymerase run-off transcription. Briefly, the tRNA^Sec^ gene with a T7 RNA polymerase promoter directly upstream was amplified by PCR from a pUC19 vector using the following primers:

Forward: 5'-CCCAGTCACGACGTTGTAAAACG -3’

Reverse: 5’-TGGCGCCCGAAAGGTGGAAT-3’

For Mutant 3 and 5 tRNA^Sec^ variants, the following *reverse* primers were used for PCR:

Mutant 3: 5’-TGGCGCCCGAAGGTGGGAATTGAACCCACT-3’

Mutant 5: 5’-TGGCGCCCGAGGTGGGAATTGAACCCACTC-3’

After verifying the template sequence by Sanger sequencing, the transcription reaction was performed at +37 °C for 4-5 h in 40 mM Tris, pH 8.0, 22 mM MgCl_2_, 2 mM spermidine, 0.01% (v/v) Triton X-100, 50 mg/mL PEG 8,000, 50 µg/mL BSA, 10 mM DTT, 10 mM GMP, 4 mM of each rNTP (ATP, GTP, CTP, and UTP), 62.5-70 μL/mL of the tRNA^Sec^ PCR product (unpurified), and 50 μg/mL of T7 RNA polymerase. Prior to purification, precipitated magnesium pyrophosphate was pelleted by centrifugation and removed. The cleared reaction was loaded onto a Resource-Q column (Cytiva), and tRNA^Sec^ was purified using a linear gradient of NaCl (0.4-0.7 M) in 20 mM Tris, pH 8.0. Following elution, the human tRNA^Sec^ was further purified on a S75 16/60 Superdex SEC column (Cytiva) equilibrated in 20 mM HEPES, pH 8.0 and 150 mM NaCl. The eluted tRNA^Sec^ was concentrated to ~300 µM, filtered through a 0.22 μm filter, flash-frozen in liquid nitrogen, and stored at -80 °C.

**Growth curves for Δ*selA* JS2(DE3) strains.** *E. coli* strains possessing either pET15b and pACYCDuet-1 empty vectors, pET15b and pACYCDuet-1-Myc-PSTK, or pET15b-WT SepSecS and pACYCDuet-1-Myc-PSTK were grown aerobically for 16 h at +37°C in LB broth supplemented with 1% (w/v) glucose, carbenicillin (100 µg/mL), and chloramphenicol (34 µg/mL). OD_600_ was determined for each overnight culture and used to calculate the cell density. In the anerobic chamber (Type A vinyl 110V, Coy Lab Products), 25 mL of LB supplemented with 10 µM IPTG, 1 µM Na_2_MoO_4_, 1 µM Na_2_SeO_3_, 50 mM HCOONa, and 0.5% (w/v) glucose was inoculated with 4 * 10^6^ cell/mL. Cultures were grown anaerobically with a gas mix of 90% N_2_, 5% H_2_, 5% CO_2_ for 48 h at +25°C. At each time point, a 3 mL aliquot was collected, serially diluted in sterile PBS, and plated onto LB agar plates with carbenicillin (100 µg/mL) and chloramphenicol (34 µg/mL). Growth was monitored by determining the CFU/mL of culture at 0, 2, 5, 8, 20, 24, 30, and 48 h. Each experiment was repeated in triplicate.

**Western blotting of *E. coli* complementation assay strains.** At the end of the *E. coli* complementation assay, cells from the LB agar plates onto which 250 μL of culture were spread were collected to verify SepSecS and PSTK expression during the assay. To each plate, 5 mL of sterile PBS was added and aspirated several times to dislodge cells from the plate. To determine the cell density, a small aliquot was taken and diluted 10-50-fold in PBS and the OD_600_ determined using an FLUOstar Omega Microplate Reader. For each strain, 4 * 10^9^ cells were then spun down and lysed in 400 μL B-PER (Thermo Scientific) supplemented with 50 μg/mL of lysozyme, 5 U/mL of DNase I, and a protease inhibitor tablet (Roche) according to the manufacturer’s protocol. Subsequently, the total protein was determined using the Pierce BCA assay (Thermo Scientific) and measured with the FLUOstar Omega Microplate Reader at 562 nm. For SDS-PAGE, either 10 or 20 (for the control blot) μg of total protein was loaded and resolved on 4-15% TGX gels (Bio-Rad). For subsequent immunoblotting, gels were transferred onto a Immobilon-FL 0.45 μm PVDF membrane (EMD Millipore), and protein loading was evaluated using Ponceau staining. For Li-COR western blotting, the membranes were blocked with 5% nonfat dry milk in Tris-buffered saline (20 mM Tris, pH 7.6, 150 mM NaCl, TBS) for 1 h at room temperature. Subsequently, the membrane was incubated with either a rabbit, anti-SepSecS antibody (1:2000) (Novus Biologicals, NBP2-20454) or a mouse anti-Myc antibody (1:1000) (Cell Signaling, 9B11) in blocking buffer with 0.1% Tween-20 (TBST) overnight at 4 °C. The membranes were then washed with TBST, incubated with secondary antibodies in blocking buffer in TBST for 1 h at room temperature, washed with TBST, and imaged with the Odyssey CLx Infrared Imaging System (Li-COR). Mouse and rabbit antibodies were detected using an anti-mouse, DyLight™ 680 Conjugate (1:15,000) (Cell Signaling, 5470P) or an anti-rabbit (DyLight 800 4xPEG Conjugate (1:30,000) (Cell Signaling, 5151S).

**SUPPLEMENTAL TABLES AND FIGURES**

**Figure S1. Experimentally derived SAD maps for SeMet SepSecS•tRNA^Sec^**. **(A)** Strong 7σ anomalous difference Fourier map peaks (purple mesh) reveal positions of Se sites in SeMet-derivatized SepSecS•tRNA^Sec^. **(B)** The same map, contoured at 5σ, reveals alternate conformations of the Mse423 side chain. **(C, D)** The experimental electron density map (blue mesh, 0.9σ) shows alternate conformations of tRNA^Sec^ within the same asymmetric unit. Conformers A (light green sticks, **C**) and B (dark green sticks, **D**) are highlighted against the other conformer (B or A, respectively) which is colored gray. **(E)** Closeup view of the experimental electron density map (blue mesh, 1.8σ) covering the PLP co-factor in the active site of the non-catalytic protomer (red and pink sticks).

**Figure S2. Structural overlays illustrate that holo- and tRNA-bound SepSecS adopts a similar tetrameric architecture. (A)** Superimposition of holo-SepSecS (grey cartoon) and the native SepSecS•tRNA^Sec^ complex (red cartoon) (RMSD of 0.473 Å over 12,368/13,836 atoms), (**B)** holo-SepSecS and the SeMet-SepSecS•tRNA^Sec^ (teal cartoon; RMSD of 0.498 Å over 12,118/13,708 atoms), and **(C)** of the native and SeMet-derivatized binary complexes (RMSD of 0.214 Å over 13,653/17,606 atoms).

Figure S3. Sequence alignment of archaeal and eukaryotic SepSecS. SepSecS sequences were aligned to secondary structure elements derived from either the crystal structure of human holo SepSecS (PDBID 7L1T) or the PSIPRED secondary structure prediction. PSIPRED secondary structure generally coincides with the PDB secondary structural assignments. Several strictly conserved residues are found in the P-loop (residues 96-107). Hydrophilic amino acids in the major tRNA-binding helices α1 and α14 (boxed in green) are less strictly conserved. PSIPRED secondary structure numbering has been removed for the sake of clarity. Residues predicted to interact with the nitrogenous bases of tRNA^Sec^ are denoted with a purple star. PDB alpha helical assignment for α12 corresponds to α12 and α13. Thus, numbering following α12 is α(n+1). Correct secondary structural assignment can be found in Supplementary Figure S4.

**Figure S4. Revised secondary structure topology for human SepSecS.** Secondary structure assignments are represented as red cylinders (α-helices), blue arrows ( β-strands) and black lines (loops). The newly discovered C-terminal helix (α16) is purple.

**Figure S5. Citrate binds to the P-loop in the binary complex crystal structure.** The electrostatic surface potential of the SepSecS•tRNA^Sec^ binary complex shows that the active site of SepSecS is strongly positively charged and compatible with large, negatively charged anions, such as citrate (CIT; grey ball-and-sticks). The positive charge of the active site is complementary to PLP (blue ball-and-sticks), the CCA-end of tRNA^Sec^, and reaction substrates (*i.e.*, phosphoseryl and selenophosphate) and products (*i.e.*, Sec and phosphate).

Figure S6. Binding of α1 and α14 SepSecS mutants to WT tRNA^Sec^. (A-B) The α1 mutants R26A and K38M exhibit decreased binding affinity compared to the WT enzyme, while R33A is a stronger binder of tRNA^Sec^. (C) The binding curves of α14 mutants show that R398A and Q399A exhibit reduced affinity, whereas F396V and T397V bind tRNA^Sec^ with higher affinity compared to the WT enzyme. Data points are omitted for clarity. Raw MST-generated binding curves and corresponding fitting statistics are shown in Supplementary Figures S6 and S7.

**Figure S7.** **MST-generated binding curves of α1 SepSecS mutants with WT tRNA^Sec^.** The normalized fluorescence (F_norm_ in [‰]) obtained from each labeled mutant (5 nM) was plotted against the concentration of WT tRNA^Sec^. MST-on time was 5 s with 3-4 independent measurements per experiment; error bars represent the standard deviation. The inset tables show the derived K_d_ along with binding statistics. **(A)** R26A-hSSS; **(B)** S27A-hSSS; **(C)** H30A-hSSS; **(D)** R33A-hSSS; **(E)** E37L-hSSS; **(F)** K38M-hSSS. *RA, response amplitude; S/N, signal-to-noise; Std. Error of Reg., standard error of regression; Red. χ^2^, reduced χ^2^.*

Figure S8. MST-generated binding curves of α14 SepSecS mutants with WT tRNA^Sec^. The normalized fluorescence (F_norm_ in [‰]) obtained from each labeled mutant (5 nM) was plotted against the concentration of WT tRNA^Sec^. MST-on time was 5 s with 4-6 independent measurements per experiment; error bars represent the standard deviation. The inset table within each plot reports the derived K_d_ along with binding statistics for each mutant’s interaction with WT tRNA^Sec^. (A) S393A-hSSS; (B) F396V-hSSS; (C) T397V-hSSS; (D) R398A-hSS (E) R398E-hSSS (a non-binder); (F) Q399A-hSSS. *RA, response amplitude; S/N, signal-to-noise; Std. Error of Reg., standard error of regression; Red. Χ^2^, reduced χ^2^.*

**Figure S9. The van’t Hoff data and plot for the high-affinity R33A mutant. (A)** The normalized fluorescence (F_norm_ in [‰]) obtained from the labeled, R33A mutant (5 nM) was plotted against the concentration of WT tRNA^Sec^. Analysis of all mutants used an MST-on time of 5 s with n = 6 independent measurements; error bars represent the standard deviation. **(B)** MST-generated binding curve for the R33A-SepSecS:tRNA^Sec^ interaction monitored over the temperature range of +24-34 °C. Increasing temperature results in a decreased binding affinity. Data points and error bars removed for clarity. **(C)** van’t Hoff plot of the MST-generated K_a_ for each temperature.

**Figure S10. The van’t Hoff data and plot for α14 high-affinity mutants.** **(A-B)** Raw fluorescence data for the MST experiment. The normalized fluorescence (F_norm_ in [‰]) from 5 nM of the labeled mutant was plotted against the concentration of WT tRNA^Sec^. Analysis of all mutants used an MST-on time of 5 s with n = 6 independent measurements; error bars represent the standard deviation. **(C-D)** MST-generated binding curves, shown as fraction bound vs. [tRNA^Sec^], shows that increasing temperature results in decreased affinity. Data points and error bars removed for clarity. **(E-F)** van’t Hoff plot of the MST-generated K_a_ for each temperature point.

**Figure S11. SepSecS and PSTK co-expression confers a growth advantage to the Δ*selA* JS2(DE3) strain.** The growth curve of strains expressing either empty vectors (EV + EV; empty black circles and black line;), PSTK alone (EV + PSTK; empty red squares and red line), or both WT SepSecS and PSTK (WT SSS + PSTK; filled green triangles and green line) under anaerobic conditions. Co-expression of SepSecS and PSTK allows the strain to reach higher cell density, likely due to the host’s ability to utilize formate as an energy source.

**Figure S12. Δ*selA* JS2(DE3) strains express SepSecS and PSTK.** Western blots confirm expression of SepSecS and PSTK in control strains **(A),** and strains harboring either α1 helix **(B)** or α14 helix mutants **(C)**.

**Figure S13. Secondary structure diagrams of human tRNA^Sec^ variants. (A)** Nucleotides mutated or replaced with elements from tRNA^Ser^ are denoted with boxes, while an arrow points to a position where the G52:C62 base pair is inserted in the TΨC arm. **(B-D)** Excerpts of secondary structure diagrams showing altered elements in the corresponding variants. The remainder of each construct is the same as in WT tRNA^Sec^. Diagrams above each tRNA construct indicate the predicted fold and the number of base pairs in the acceptor- (pink), variable- (yellow), and TΨC-(cyan) arms. The anticodon and D-arms are gray and green, respectively, while the CCA-end is not shown for brevity.

**Figure S14. MST-generated binding curves of tRNA^Sec^ mutants with WT SepSecS.** The normalized fluorescence (F_norm_ in [‰]) obtained from WT SepSecS (5 nM) was plotted against the tRNA concentration. MST-on time was 5 s with 4-6 independent measurements per experiment; error bars represent the standard deviation. The inset table within each plot contains K_d_ values along with binding statistics for each interaction. **(A)** vSer-tRNA^Sec^; **(B)** ΔVar-tRNA^Sec^; **(C)** Mut3-tRNA^Sec^; **(D)** Mut5-tRNA^Sec^; **(E)** comparison of variant binding curves to WT tRNA^Sec^ (dashed blue curve). Compared to the WT interaction, vSer and Mut3 exhibited decreased affinity while ΔVar resembled the WT interaction. *RA, response amplitude; S/N, signal-to-noise; Std. Error of Reg., standard error of regression; Red. Χ^2^, reduced χ^2^.*

**Table S1. Data collection and refinement statistics.**

|  | **Holo SepSecS** | **SepSecS**•**tRNA^Sec^** | **SeMet-SepSecS**•**tRNA^Sec^** |
| --- | --- | --- | --- |
| **PDB ID** | **7L1T** | **7MDL** | **8G9Z** |
| **Data Collection** |  |  |  |
| Space group | P622 | P3_1_12 | P3_1_12 |
| Unit cell dimensions:  a=b, c (Å)  α, β, γ (°) | 154, 154, 76  γ=120 | 167, 167, 240  γ=120 | 167, 167, 239  γ=120 |
| Resolution range (Å) | 50.00 – 2.25 (2.29-2.25) | 50.00 – 2.32  (2.36-2.32) | 50.00 – 2.07 (2.11-2.07) |
| Unique reflections* | 25,765 (1,257) | 165,776 (8,185) | 456,255 (11,482) |
| R_meas_ (%) | 9.9 | 14.4 | 11.8 |
| I/ σ(I) * | 4.5 (0.7) | 7.9 (1.0) | 4.3 (1.1) |
| Completeness (%)* | 99.9 (100.0) | 99.9 (99.6) | 100.0 (100.0) |
| Redundancy*  CC_1/2_  Figure of merit | 8.5 (8.9)  (0.169)  N/A | 7.1 (4.5)  0.994 (0.403)  N/A | 10.7 (10.6)  0.996 (0.337)  0.316 |
| **Refinement** |  |  |  |
| Resolution range (Å) | 30.64 – 2.25  (2.31 – 2.25) | 41.6 – 2.32  (2.38 – 2.32) | 34.25 – 2.07  (2.09 – 2.07) |
| Average B-factor: | 65.4 | 50.1 | 59.0 |
| Macromolecule | 65.4 | 50.1 | 59.4 |
| Ligands | 71.1 | 56.8 | 55.6 |
| Solvent | 61.7 | 48.7 | 54.8 |
| Number of residues:  Protein  Nucleic acid | 457  N/A | 1,847  89 | 1,853  89 |
| Non-hydrogen atoms: | 3588 | 19,301 | 19,953 |
| Macromolecule | 3,472 | 17,877 | 18,524 |
| Ligands | 20 | 112 |  |
| Solvent | 96 | 1,311 | 1,377 |
| R_work_* | 19.2 (38.8) | 17.6 (29.5) | 17.7 (35.7) |
| R_free_* | 23.0 (39.8) | 20.3 (32.9) | 19.0 (39.6) |
| RMS deviations:  Bonds (Å) | 0.002 | 0.002 | 0.002 |
| Angles (°) | 0.47 | 0.62 | 0.53 |
| Ramachandran plot:  Favored (%) | 96.7 | 96.8 | 97.6 |
| Allowed (%) | 3.3 | 3.2 | 2.3 |

*Statistics for the highest-resolution shell are shown in parentheses.

**Table S2. Statistics for the SepSecS mutant unfolding profiles.**

| **Sample** | **T_i_***^a^* | **Initial**  **Ratio***^b^* | **Δ Ratio***^c^* |
| --- | --- | --- | --- |
| **WT** | 75.263 ± 0.001 | 0.550 ± 0.001 | 0.247 ± 0.000 |
| **R26A** | 73.944± 0.033 | 0.546 ± 0.001 | 0.249 ± 0.003 |
| **S27A** | 76.957 ± 0.000 | 0.548± 0.001 | 0.246 ± 0.003 |
| **H30A** | 76.587± 0.016 | 0.547± 0.000 | 0.245± 0.001 |
| **R33A** | 75.034 ± 0.092 | 0.543± 0.000 | 0.255± 0.000 |
| **E37L** | 75.396 ± 0.015 | 0.551± 0.001 | 0.253± 0.003 |
| **K38M** | 73.233 ± 0.017 | 0.549 ± 0.000 | 0.253± 0.002 |
| **S393A** | 75.789 ± 0.274 | 0.552 ± 0.000 | 0.245 ± 0.000 |
| **F396V** | 74.032 ± 0.027 | 0.547 ± 0.000 | 0.246 ± 0.001 |
| **T397V** | 74.437 ± 0.022 | 0.569 ± 0.009 | 0.236 ± 0.001 |
| **R398A** | 76.007± 0.008 | 0.545 ± 0.000 | 0.246 ± 0.000 |
| **R398E** | 76.373 ± 0.086 | 0.547 ± 0.000 | 0.246 ± 0.000 |
| **Q399A** | 73.828 ± 0.000 | 0.545± 0.000 | 0.249 ± 0.000 |

*^a.^*T_i_ is the inflection temperature of the unfolding transition in the signal of the 350/330 nm ratio.

*^b.^* Initial Ratio is the value of the ratio of 350/330 nm at the beginning of the measurement*.*

*^c.^*Δ Ratio is the difference between the ratio at the beginning and at the end of the thermal profile.

**References**

1. Palioura,S., Sherrer,R.L., Steitz,T.A., Söll,D. and Simonović,M. (2009) The Human SepSecS-tRNA^Sec^ Complex Reveals the Mechanism of Selenocysteine Formation. *Science*, **325**, 321–325.

2. Walden,H. (2010) Selenium incorporation using recombinant techniques. *Acta Cryst D*, **66**, 352–357.
